# Mango mesocarp fibers are a continuum of fibrous endocarp structures that are co-lignified during seed coat hardening

**DOI:** 10.3389/fpls.2026.1874130

**Published:** 2026-07-23

**Authors:** Mazal Ish-Shalom, Adi Doron-Faigenboim, Hanita Zemach, Amir Sherman, Yuval Cohen

**Affiliations:** Institute of Plant Sciences, Agricultural Research Organization - The Volcani Institute, Rishon LeZion, Israel

**Keywords:** endocarp, fibers, lignin, *Mangifera indica*, mango, mesocarp, secondary cell wall

## Abstract

Mango fruit is a drupe containing a single seed enclosed in a hard, fibrous endocarp surrounded by an edible fleshy mesocarp which contains significant fibrous structures. We performed morphological, histological and gene expression analyses of mango fibers during fruit maturation and endocarp hardening of “fibrous” and “non-fibrous” accessions, to characterize the developmental process and explore the molecular mechanism of fiber formation and hardening. All studied accessions contain fibers, but they differ in their length and rigidity. Mesocarp fibers develop as braid-like fibrous structures of the endocarp, early in fruit development and lignified in parallel to the hardening of the endocarp fibers during fruit maturation and seed hardening. Polyphenol and lignin biosynthesis pathways and secondary cell wall deposition are the major processes involved in fiber hardening in both endocarp and mesocarp fruit tissues. Mango mesocarp fibers are a structural continuum of the fibrous endocarp. Lignification plays a major role in fibrousness. The resemblance to fibrous mesocarp tissues of other drupe species suggests a common mechanism with both evolutionary and horticultural importance. The results offer new insight into the evolution of fruit structure in mango and may provide a path to breed “freestone” varieties with reduced fibrousness.

## 1 Introduction

Plant fibers are characterized by long cell structures, having a thickened secondary cell wall that provides mechanical support to organs and the entire plant (Gorshkova et al., 2012). Fibers can be part of both the primary and secondary plant body, and may be associated with xylem and phloem tissues (Lev-Yadun, 2010). Fibers may appear as single cells or as bundles and are present in many different organs, including roots, stems, leaves and fruits (Gorshkova et al., 2012; Célino et al., 2014). Plant fibers have well-developed secondary cell wall composed of three main components: cellulose, hemicelluloses (xylan and glucomannan), and lignin (Gorshkova et al., 2012; Zhong et al., 2019). The secondary cell wall is formed by successive deposition of cellulose layers, while lignin polymerization and cross-linking to polysaccharides strengthen and hold the cell wall components.

Lignin is abundant in the secondary cell walls of plants and confers structural support, mechanical strength, rigidity, pathogen defense and impermeability (Vanholme et al., 2019; Zhong et al., 2019; Emonet and Hay, 2022). In different plant species, lignified tissues enable diverse functions depending on tissue rigidity (Emonet and Hay, 2022). It is a complex polyphenolic polymer derived from the oxidative radical coupling of three monolignols—p-coumaryl alcohol, coniferyl alcohol and sinapyl alcohol, which produce respectively, p-hydroxypheny (H), guaiacyl (G), and syringyl (S) lignin units, during polymer formation in a process of lignification (Boerjan et al., 2003). Monolignols are synthesized from phenylalanine via the phenylpropanoid pathway in the cytosol and then transported to the cell wall, where they undergo oxidation and polymerization (Boerjan et al., 2003; Vanholme et al., 2019).

Lignin is essential for plant growth and development and affects the quality of edible fruits and vegetables. Many studies have attempted to elucidate the lignification process in fruits. Lignification and secondary cell wall thickening are major processes in endocarp hardening in stone fruits and other fruit crops (Dardick et al., 2010; Dardick and Callahan, 2014; Mao et al., 2024). Lignification also occurs in edible plant organs, such as stone cells, a type of sclerenchyma in pear parenchyma, which form through the deposition of lignin on cell walls following secondary thickening (Tao et al., 2009; Cai et al., 2010).

In some drupe fruits (fleshy fruit that contain a hard pit or “stone”, enclosing a single seed, such as loquat and lychee, the endocarp remains thin with low lignification (Khan et al., 2022) and the stone is free from the flesh. In other drupe fruits, including jujube (Mao et al., 2024), olive, oil palm, species of Anacardiaceae (mango, pistachio and morulla) and many Rosaceae fruits of the *Prunus* genus (cherry, peach and plum) (Dardick and Callahan, 2014; Khan et al., 2022) the stone strongly adheres to the flesh. A stoneless mutant in plums has been documented (Burbank, 1914; Callahan et al., 2015). Stoneless cultivars of Chinese Jujube have also been developed and characterized (Mao et al., 2024). Similarly, in oil palm, a single mutation in the *SHELL* gene, which is homologous to Arabidopsis *SEEDSTICK*, an *AGAMOUS*-like gene, controls endocarp thickness, and mutations can completely eliminate the endocarp (Singh et al., 2013). Another important trait related to endocarp-mesocarp boundaries is the Clingstone - Freestone phenotypes of peach and plums: Clingstone, where the flesh strongly adheres to the stone, and Freestone were the endocarp is not attached to the mesocarp (Wang et al., 2024). As these traits affects eating quality, they are important breeding targets.

Mango (*Mangifera indica* L.), a fruit tree of the family Anacardiaceae, is one of the most important fruit crops in the world, with global annual production of more than 60 million tons (FAOSTAT, 2024). The fruit is a drupe containing a single large seed enclosed in a hard, fibrous endocarp, surrounded by the edible fleshy mesocarp (Mukherjee and Litz, 2009). Fibrousness in the mango mesocarp is a major quality trait. Fibers are present in all mango cultivars, but some are considered more fibrous than others. Our initial characterization of mango fibers suggested that two main types of fibers can be detected: thicker fibers, derived from the fruit stalk, surrounding the periphery of the mesocarp just under the fruit peel, and fibers that emerge from the endocarp towards and into the fleshy mesocarp. Usually, fibers of the latter type are more densely formed on the basal ventral side of the fruit (Ish-Shalom et al., 2023). Higher lignin content was found in mesocarp of a more fibrous cultivar, ‘Tommy Atkins’ compared with the less fibrous ‘Kent’ (Brito and Vaillant, 2012), suggesting that lignin deposition in the fibers may influence their hardening. However, the processes and genes contributing to fibers formation in mango remain unstudied.

Until recently, mango was an orphan crop, with only a few studies on fruit development, and very little molecular information. Several studies have focused on creating genomic tools for mango by providing transcriptome data (Azim et al., 2014; Dautt-Castro et al., 2015; Sherman et al., 2015; Tafolla-Arellano et al., 2017), genetic maps (Luo et al., 2016; Kuhn et al., 2017; Srivastav et al., 2023), and several draft genome sequences (Wang et al., 2020; Bally et al., 2021; Ma et al., 2021; Wijesundara et al., 2024). This new genomic data can now enable exploring genes involved in fiber formation during fruit development in mangoes. In this study, we characterized fiber formation in mango fruits from some “fibrous” and “non- fibrous” accessions during fruit development and seed hardening. Our hypothesis was that there is a direct connection between the mesocarp fibers and the endocarp tissue. We aimed to shed light on the molecular processes leading to fiber formation and lignin accumulation in the mesocarp.

## 2 Materials and methods

### 2.1 Plant material

Mango cultivars ‘Omer’, ‘Shelly’, the fibrous line BD 40/92 (a fibrous line of the Israeli breeding program) (all grafted on 13/1 rootstock), and 13/1 rootstock itself, grown in the experimental orchard of the Agricultural Research Organization, the Volcani Institute in Rishon LeZion, Israel, were used in this study. Fruits (at least five fruits per tree, from two trees per accession), were sampled at five distinct time points, at approximately 75 to 135 days after flowering (DAF). These sampling intervals represent crucial stages of fruitlet development: from young fruit, prior to, during and after endocarp hardening, extending up to full fruit ripening, across the summers of 2017 and 2018. Upon sampling, fruits were sliced longitudinally into halves and mesocarp and endocarp tissues were collected from the ventral shoulder, at the basal part of the fruit (**Supplementary Figure 1**). Samples were collected and processed immediately for histological analysis. For molecular analysis, tissue samples of endocarp and mesocarp were compiled at three specific developmental stages: before endocarp hardening (June, 86 DAF), during hardening (July, 106 DAF) and post hardening (August, 134 DAF). Tissues were immediately frozen in liquid nitrogen and kept at -80 °C until use.

For whole-fruit imaging of fibers, ripened fruits were washed under running water, and the fibers attached to the seeds were gently separated from the peel and from the mesocarp tissue (Ish-Shalom et al., 2023). Samples were then dipped in a water tank to enable spread the fibers for documentation.

### 2.2 Whole fruit lignin staining

Mango fruits collected during development and ripening were cut longitudinally. Lignin containing elements were visualized using phloroglucinol-HCl, according to Callahan et al. (2009) and Dardick et al. (2010). Cut fruits were dipped in a phloroglucinol solution [1% (w/v) phloroglucinol, 12% HCl (v/v), and 85% ethanol (v/v)] for 1 h. The fruits were then rinsed in 95% ethanol (v/v) and photographed.

### 2.3 Histology and microscopy

Fruit samples (taken from the ventral basal fruit side near the fruit stalk) containing the mesocarp and the endocarp regions were collected during development, and fixed in FAA solution [10% formaldehyde: 5% acetic acid: 50% ethanol: 35% H_2_O (v/v)]. Samples kept in FAA were gradually dehydrated in ethanol (50, 70, 90, 95, and 100%), followed by gradual replacement with HistoClear, and paraffin embedment (using Paraplast Plus – Tissue Embedding Medium, Leica). Samples were sectioned using a Leica RM2245 microtome and stained with Safranin/Fast Green, according to Ruzin (1999). Slides were examined with a light microscope (ECLIPSE Ni-E, Nikon), photographed with a Nikon DS-Fi1 digital camera and analyzed using Nikon NIS-elements program.

### 2.4 RNA extraction

Total RNA was isolated from 2 g of mango fruit mesocarp or endocarp tissue ground in liquid nitrogen, using a cetyltrimethylammonium bromide (CTAB)-based method adapted from Chang et al. (1993). Residual DNA contamination was digested using 1 U of RQ1 RNase-free DNase (Promega) in the presence of 40 U of RiboLock RNase Inhibitor (Thermo Fisher Scientific) for 30 min at 37 °C. The RNA was subsequently re-extracted using phenol:chloroform:isoamyl alcohol (25:24:1, v/v/v), precipitated, and resuspended in ultrapure water. RNA quality and purity were assessed using a NanoDrop™ 1000 Spectrophotometer (Thermo Fisher Scientific) and verified via agarose gel electrophoresis.

### 2.5 RNA Sequencing and bioinformatic analysis

Next-generation sequencing was performed on samples taken from two fibrous accessions (13/1 rootstock and ‘BD40/92’) and from two non-fibrous accessions (‘Omer’ and ‘Shelly’). Endocarp and mesocarp tissues from fruits before endocarp hardening (June), during hardening (July) and after hardening (August) were used. A total of four biological samples of fibrous accessions (from several fruits of two trees of 13/1 and from two trees of BD40/92 line) and four biological samples of fruits of non-fibrous accessions (from two trees of ‘Omer’ and two trees of ‘Shelly’) were sequenced (48 samples in total) using TruSeq RNA Library v2 construction + NovaSeq Illumina 2x100bp sequencing (Macrogen, South Korea) generating approximately 41 million reads per sample.

Raw reads were subjected to a cleaning procedure using the FASTX Toolkit (http://hannonlab.cshl.edu/fastx_toolkit/index.html, version 0.0.13.2), which included (1) trimming read-end nucleotides with quality scores < 30 using fastq_quality_trimmer; (2) removing read pairs if either one had less than 70% base pairs with quality score ≤ 30 using fastq_quality_filter. The SortMeRNA tool was used to filter out rRNA. Clean reads were mapped to the reference ‘Tommy Atkins’ mango genome (Bally et al., 2021) using TopHat2 software (Kim et al., 2013, v. 2.1) with an average mapping rate of 89.3%. The Cufflinks suite of tools (Trapnell et al., 2012, v. 2.2) was used for gene abundance estimation combined with gene model defined by the cufflink-cuffmerge-cuffquant pipeline. Heatmap visualization and principal component analysis (PCA) were performed using R Bioconductor (Gentleman et al., 2004) and ClustVis tool (Metsalu and Vilo, 2015). Gene expression values were computed as fragments per kilobase of transcript per million mapped reads (FPKM). Differential expression analysis was performed using the DESeq2 R package (Love et al., 2014) based on the raw count data calculated using Tophat mapping software and cuffqunat tool. Genes with an adjusted p-value of ≤0.05 were considered differentially expressed. Venn diagrams were generated using the online tool at bioinformatics.psb.ugent.be/webtools/Venn/.

The gene sequences were used as query terms for a search of the NCBI non-redundant (nr) protein database that was carried out with the DIAMOND program (Buchfink et al., 2015). The search results were imported into Blast2GO version 4.0 for gene ontology (GO) annotation. Gene ontology enrichment analysis was carried out using Blast2GO program based on Fisher’s Exact Test with multiple testing correction of false discovery rate (FDR). KOBAS 3.0 tool (http://bioinfo.org/kobas) was used to identify statistically enriched pathways of differentially expressed genes in both KEGG and GO categories.

### 2.6 Statistical analysis

Data were processed using JMP software (Ver.14). Nested analysis of variance (ANOVA) was used to compare means with Tukey-Kramer HSD test.

## 3 Results

### 3.1 Morphological assessment of the mango fibers

Selected ripened fruits from various mango accessions were gently mashed and softened under running water until the peel was easily removed, while preserving the fibers and gently separating them from the mesocarp tissue (**Figure 1**). Fruits contain two types of fibers: (1) long and relatively thick ones, deriving directly from the fruit stalk; these are localized on the mesocarp periphery, just below the peel (**Figures 1B, C**). These long peripheral fibers are most probably vascular elements, nourishing the growing fruit; (2) Shorter and thinner fibers emerging directly from the endocarp and penetrating the mesocarp, sometimes forming dense fiber formations within the mesocarp (**Figures 1B, D**). This pattern was observed in all tested accessions. Comparison of the shorter fibers in two fibrous lines, 13/1 rootstock (**Figure 1E**) and BD40/92 (**Figure 1F**)) and two non-fibrous cultivars, ‘Omer’ (**Figure 1G**) and ‘Shelly’ (**Figure 1H**) suggests that they are harder in fibrous fruits, and cross the entire mesocarp, while in the non-fibrous accessions they are shorter, gentle and softer.

**Figure 1:**
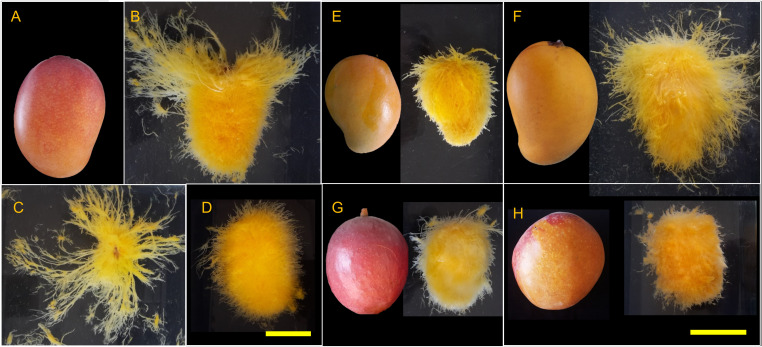
Organization of fibers in the mango mesocarp. **(A)** Whole fruit of the mango cultivar ‘Tommy Atkins’. **(B)** Representation of fibers in the fruit. Two types of fibers are presented: long and thick fibers derived from the fruit stalk and short fibers emerging directly from the endocarp. **(C)** the long and peripheral fibers and **(D)** short fibers emerging from the endocarp, after their separation. The shorter fibers emerging from the endocarp (following the removal of the peripheral fibers) of fibrous fruits of **(E)** 13/1 and **(F)** BD40/92 and the non-fibrous cultivars **(G)** ‘Omer’ and **(H)** ‘Shelly’ aside with the whole fruit are documented. In all fruits, the fibers were gently separated under water from the fruit peel and flesh, then dipped in water to spread the fibers for documentation. Bars represent 5 cm scales.

Lignification of the short fibers and their connection to the endocarp was studied in ripened fruits that were cut lengthwise and stained with phloroglucinol. In fibrous mango accessions stronger lignin staining was detected in the mesocarp compared with non- fibrous accessions (**Figures 2A, B**). While stained fibers were detected in all the mesocarp regions, more fibers were detected at the basal and ventral side (**Figure 2B**).

**Figure 2:**
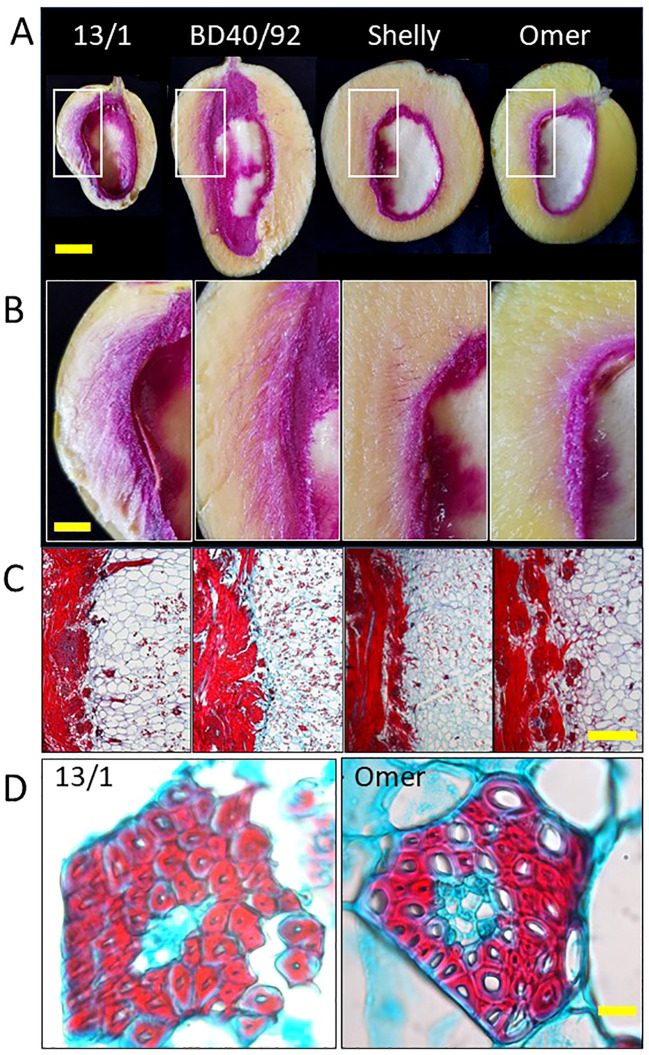
Lignification of the endocarp and the fibers penetrating the mesocarp in fibrous (‘13/1’ and ‘BD40/92’) and non-fibrous cultivars (‘Omer’ and ‘Shelly’). **(A)** Whole ripened fruits, were cut longitudinally and stained with phloroglucinol. **(B)** Magnified area at the basal ventral side of the fruits in the rectangles of **(A)** respectively; **(C)** Low-magnification longitudinal histological sections of the border between the endocarp (left) and the mesocarp (right) tissues of fibrous accessions and non-fibrous cultivars. **(D)** Cross section through individual mesocarp fibers in ‘13/1’ and ‘Omer’. Samples were fixed, embedded in Paraffin and sections were stained with Safranin-Fast green. Bars represent 3 cm **(A)**,1 cm **(B)**, 500 µm **(C)** and 50 µm for **(D)** respectively.

Longitudinal sections through the endocarp and mesocarp revealed a unique structure of the fibers, forming bundles, associated to complex “braid” like structures at the outer endocarp; some penetrate into the mesocarp tissue (**Figure 2C**). These are detected in both fibrous and non - fibrous cultivars. Cross-sections of the fibers revealed bundles of cells, with well-defined, highly lignified cell walls boundaries surrounding a central zone (**Figure 2D**). Comparing this circumferential Safranin red staining in 13/1 cultivar to ‘Omer’ revealed a visibly higher staining intensity within the cell walls of the fibrous 13/1 fiber bundles, reflecting a more pronounced accumulation of lignified structural components.

Lignin deposition in longitudinal sections of whole fruits of ‘13/1’, was studied during fruit development (**Figure 3A**). The process started approximately 80 DAF. Comparison of endocarp hardening and lignification in 13/1 and the non-fibrous ‘Shelly’ suggests that the processes occur in parallel in the two cultivars, but in non-fibrous cultivar ‘Shelly’ it occurred to a lesser extent (**Figure 3B**). We further explored the process of fibers hardening in the mesocarp and their connection to the endocarp during development of the representative fibrous accession, 13/1 by histology (**Figures 3C, D**). At early stages of young fruit, the endocarp did not show lignification, as revealed by Safranin-Fast Green staining. At 86 DAF, the fibrous “braids” of the endocarp and the fibrous bundles in the mesocarp were specifically stained, suggesting localized lignification. In ripen fruits, strong red staining of the bundles was detected, indicating gradual process of lignification and hardening. The lignification of the fibers in the mesocarp was concomitant with that of the complex bundle structures in the endocarp. The parallel gradual staining of the fibers in the mesocarp and the bundles constituting the endocarp demonstrated the continuation of these fibers directly from the endocarp bundles (**Figures 3C, D**). Moreover, the data confirm that the fibers are generated early in fruit development as extension of the endocarp bundles at early stages, before endocarp hardening and lignification (**Figure 3D**).

**Figure 3:**
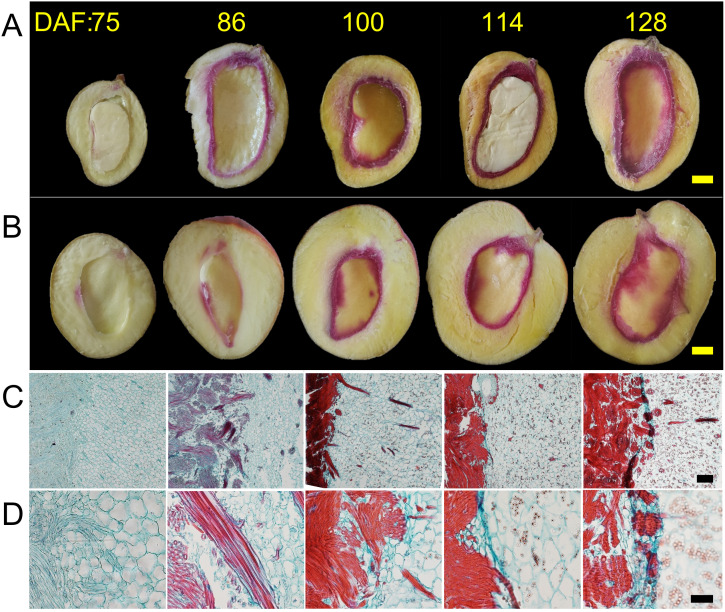
Characterization of fiber developmental and endocarp hardening in the fruit during development and ripening. Longitudinal sections of fruits of the fibrous 13/1 rootstock **(A)** and the non-fibrous cultivar ‘Shelly’ **(B)** at different developmental stages stained with phloroglucinol. **(C, D)** Histological sections at the border between the endocarp (left) and the mesocarp (right) of 13/1 fruits presented in the same developmental stages in lower **(C)** and higher **(C)** magnifications. Samples were fixed, embedded in Paraffin and sections were stained with Safranin-Fast Green. DAF – Days after full bloom. Scale bars 1 cm **(a)**, 200 µm **(b)** and 50 µm **(c)** respectively.

### 3.2 Characterization of molecular processes related to fiber’s hardening

To further explore the hardening and lignification processes in the mesocarp fibers and their relation to the fibrous structures constituting the endocarp, transcriptomic analysis was performed on endocarp and mesocarp tissues of the two fibrous accessions (13/1 rootstock and ‘BD40/92’) and the two non-fibrous cultivars (‘Omer’ and ‘Shelly’) in three developmental stages during endocarp lignification: before endocarp hardening (June, 86 DAF), during hardening (July, 106 DAF) and post hardening (August, 134 DAF). Approximately 41 million reads per sample were generate of which 84.7 – 90.8% mapped to the ‘Tommy Atkins’ mango genome draft (Bally et al., 2021) (**Supplementary Table 1**). Gene expression diversity in mesocarp and endocarp tissues during seed hardening was examined by principal component analysis (PCA) (**Figure 4A**). Both the first and second principal components and the second and third dimensions indicated a general separation between mesocarp and endocarp tissues (represented by full and open symbols in all four accessions respectively). As the seed hardens, the entire transcriptome undergoes a massive, programmed shift that is common across all accessions and both tissue types. Before seed hardening, the endocarp and mesocarp are transcriptomically closer together, but as time passes, they drift further apart vertically, suggesting the tissues become more specialized as hardening progresses. In addition, the second and third dimensions revealed separation between fibrous and non-fibrous accessions as well as distinct clustering of the endocarp and mesocarp tissues, suggesting that unique and different processes occur in each tissue type.

**Figure 4:**
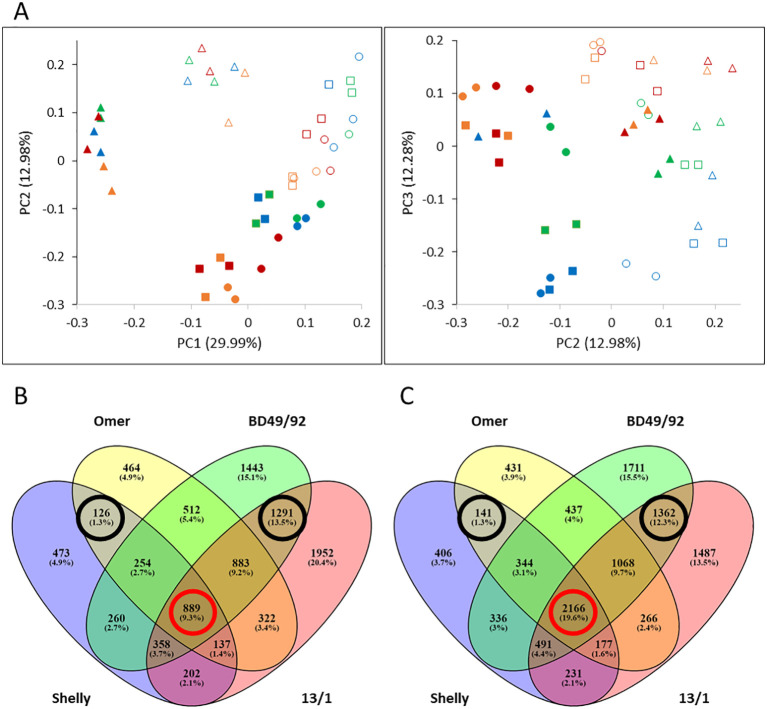
Deferential gene expression analysis in mesocarp and endocarp tissues of two fibrous accessions (‘13/1’ and ‘BD49/92’) and two none-fibrous accessions (‘Shelly’ and ‘Omer’) during seed hardening and fruit development. **(A)** Principal component analysis (PCA) of gene expression patterns in endocarp and mesocarp tissues during seed hardening. Fibrous lines ‘13/1’ and ‘BD49/92’ are marked in blue and green, and none-fibrous accessions ‘Shelly’ and ‘Omer’ are marked in red and orange respectively. Endocarp and mesocarp tissues are represented as filled and open symbols respectively. Sampling date is represented as triangles (29/6), squares (24/7) and circles (21/8). **(B, C)** Differentially expressed genes in the mesocarp **(B)** and in the endocarp **(C)** presented as a Venn diagrams. Genes common for both mesocarp and endocarp tissues across all cultivars are marked by red circles and genes specific to the two fibrous or the two non-fibrous accessions are marked by black circles.

Differentially expressed genes (DEGs) were identified in the mesocarp or endocarp of these accessions during the season (**Supplementary Figure 2**). 16,000 genes were mapped using blast to known sequences in KEGG and around 13,000 DEGs were annotated. KAAS analysis showed the involvement of these genes in selected metabolic/biochemical pathways. In the two non–fibrous cultivars (‘Omer’ and ‘Shelly’), 126 and 141 DEGs were specific to the mesocarp and the endocarp, respectively. Similarly, in the two fibrous accessions (‘BD40/92’ and ‘13/1’), 1,291 and 1,362 DEGs were specific to the mesocarp and endocarp, respectively (**Figures 4B, C**, black circles). In addition, 889 and 2,166 DEGs showed similar expression patterns across all four accessions (both fibrous and non-fibrous) in the mesocarp and at the endocarp, respectively (**Figures 4B, C**, red circles), among which 492 DEGs were common to both tissues.

We followed two approaches to analyze genes related to mesocarp fibers: First, to identify processes associated with both endocarp and mesocarp fiber formation, we focused on DEGs that are present in both mesocarp and endocarp tissues of both fibrous and non-fibrous accessions. In the second approach, we examined genes specific to the two fibrous versus the two non-fibrous accessions, aiming to identify genes specifically associated with highly fibrous lines.

### 3.3 Differentially expressed genes with similar patterns in endocarp and mesocarp

Since hardening is detected in parallel in the mesocarp fibers and the endocarp fibrous bundles, we focused on the 492 DEGs that showed common expression patterns across all accessions (fibrous and non-fibrous) and in both tissues (**Figures 4B, C**, red circles). Cluster analysis revealed two prominent groups: the first comprising 279 DEGs with high expression during early seed hardening (June) that declined in July, and the second including 213 DEGs with elevated expression later in the hardening process (July and/or August) (**Figure 5A**).

**Figure 5:**
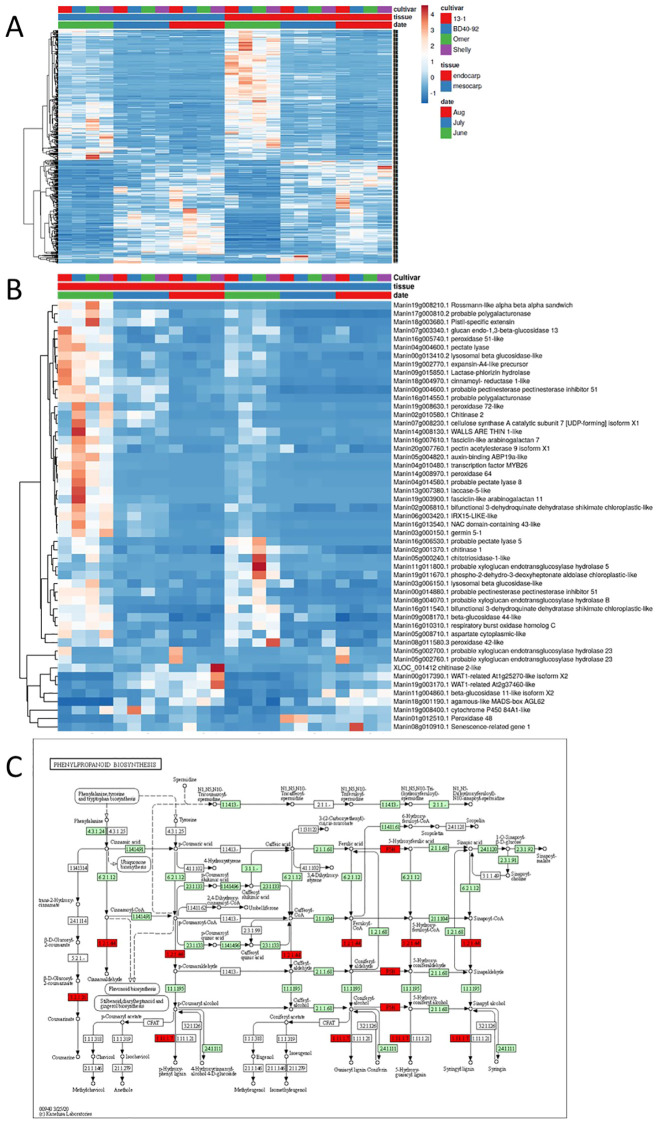
Heat map of DEGs with common expression patterns in all accessions (fibrous and non-fibrous) and in both mesocarp and endocarp tissues during fruit development tissues performed by cluster analysis. **(A)**. Expression profiles of 492 DEGs showing shared patterns across accessions and tissues. **(B)** Subset of 51 DEGs associated with secondary cell wall metabolism, phenylpropanoid biosynthesis, and related processes. Samples are ordered by accession, tissue type (endocarp or mesocarp), and sampling time (June, July, August), as indicated in the color legend. **(C)** phenylpropanoid biosynthesis pathway updated from Kyoto Encyclopedia of Genes and Genomes (KEGG) (Kanehisa and Goto, 2000; Pathway: ko00940) showing 12 distinct enzyme activities (EC numbers marked in red): five peroxidase (PER) genes, five β- glucosidase genes, cinnamoyl- reductase 1-like (CCR1) and cytochrome P450 84A1-like (CYP84A, F5H) genes. These genes shared similar expression pattern across the season in all accessions and both tissues. Enzymes marked in green represent genes identified in the RNA sequencing analysis.

Many of the DEGs, especially from cluster 1, were related to “metabolic pathways” including “biosynthesis of secondary metabolites” (**Supplementary Figure 3**). “phenylpropanoid biosynthesis” and “phenylalanine, tyrosine and tryptophan biosynthesis”, were the most significant processes (p-value ≤ 0.00001). Chorismate biosynthesis, the final product of shikimate pathway that leads to phenylalanine, tyrosine and tryptophan biosynthesis, was also enriched. Cyanoamino acid metabolism, which is connected to phenylalanine metabolism, among others, along with amino acid biosynthesis, was also detected (p-value ≤ 0.01). GO annotation reveals cell wall/external encapsulating structure and plant-type secondary cell wall biogenesis as extremely significant processes, together with cell wall organization or biogenesis and lignin metabolic process (all with p-value ≤ 0.0001). In the second cluster of the late stages expressing DEGs, we detected genes involved in energy, sugar, and fatty acid metabolism (such as pyruvate metabolism, glycolysis/gluconeogenesis and related TCA cycle). Phytohormone- and volatile-related DEGs, important for ripening, were active in the late stages of fruit maturation and ripening (July and August). KOBAS GO term analysis showed increased plastid and chloroplast activity, along with growth, reproduction, hormone response, and metabolic changes associated with fruit development and ripening.

We identified 51 DEGs related to secondary cell wall metabolism, phenylpropanoid biosynthesis and cell wall related process (all with p-value ≤ 0.01) that are common to the four accessions and both tissues. These DEGs clustered into three groups: a large cluster, subdivided into two, showing either higher expression early in endocarp hardening (June), mainly at the endocarp, or higher expression in the mesocarp and another cluster of ten genes with higher expression levels during the late hardening stage (July-August) (**Figure 5B**). Seven DEGs were specifically related to lignin metabolism processes (six biosynthesis and one catabolic genes). Ten additional DEGs (three overlapping with lignin metabolism) were related to phenylpropanoid biosynthesis. These include five peroxidase (PER) genes, five β- glucosidase genes, cinnamoyl- reductase 1-like (CCR1) and cytochrome P450 84A1-like (CYP84A, F5H) genes, all part of phenylpropanoid biosynthesis process leading to lignin synthesis (**Figure 5B**, **Supplementary Table 1**). Three additional peroxidase and 2 β- glucosidase genes were also included as they may contribute to phenylpropanoid biosynthesis. The representation of these genes in the phenylpropanoid and lignin biosynthesis pathways is presented in **Figure 5C**.

Another set of differentially expressed genes associated with secondary cell wall formation and restructuring were identified, including a Germin, gene three chitinases and Chitinase-like proteins, alongside with three *WALLS ARE THIN (WAT) 1-like* genes were detected in the dataset.

Genes related to pectin metabolism were represented by six DEGS: One pectin acetylesterase, two pectinesterase, three pectate lyase and two polygalacturonase. Furthermore, four xyloglucan endotransglucosylase hydrolase genes (XETs 23, 5, B) and “Senescence-related gene 1” (Xyloglucan:xyloglucosyl transferase) were identified, alongside a xylan biosynthesis gene, *IRX15-LIKE*.

Additional cell wall associated DEGs included three genes of the hydroxyproline-rich glycoproteins (HRGP) superfamily, specifically fasciclin-like arabinogalactan 11/7 and Pistil-specific extension. Finally, an extensin gene and an expansin gene were detected among the shared differentially expressed transcripts.

These 51 identified genes suggest the common regulatory mechanisms in lignin and secondary cell wall metabolism contribute to cell wall thickening, lignin biosynthesis, and subsequent lignin deposition, thereby driving fiber formation in both the mesocarp and endocarp tissues during the seed hardening stage in both fibrous and non-fibrous accession.

### 3.4 Differentially expressed genes with distinct patterns in fibrous vs. non-fibrous accessions

In a second approach, we analyzed genes specific to either the two fibrous or the two non-fibrous accessions. Expression patterns of the fibrous accessions (13/1 and BD40/92) were compared with those of the non-fibrous accessions (‘Omer’ and ‘Shelly’). A total of 1,291 and 1,362 common DEGs were identified in the mesocarp and in the endocarp, respectively, of the fibrous accessions and 126 and 141 common DEGs were detected in the mesocarp and in the endocarp, respectively of the non-fibrous accessions (**Figures 4B, C**, Black circles). Focusing on phenylpropanoid and lignin biosynthesis, as well as on secondary cell wall biogenesis, revealed 29 DEGs related to phenylpropanoid biosynthetic and metabolic processes and 13 DEGs associated with secondary cell wall biogenesis in the mesocarp of fibrous accessions, but not in the non-fibrous ones (**Table 1**). Heat maps showed that most of these genes displayed similar expression profiles in both mesocarp and endocarp tissues (**Figure 6**), indicating parallel processes of lignin deposition and secondary cell wall thickening during seed hardening in fibrous accessions.

**Table 1:** Differently expressed genes (DEGs) in mesocarp and endocarp tissues distinguishing fibrous and non-fibrous accessions .

	Fibrous accessions			Non-fibrous accessions		
Tissue and pathway	13/1 & BD40/92	13/1	BD40/92	Shelly & Omer	Shelly	Omer
Mesocarp						
Specific DEGs in tissue	1291	1952	1443	126	463	474
Phenylpropanoid bio./metabolic process	29	24	18	0	7	8
Plant-type secondary cell wall biogenesis & Secondary cell wall & Cell wall thickening	13	11	12	0	1	1
Endocarp						
Specific DEGs in tissue	1362	1487	1711	131	406	431
Phenylpropanoid biosynthetic/metabolic process	16	21	20	4	7	11
Plant-type secondary cell wall biogenesis & Secondary cell wall & Cell wall thickening	8	4	8	3	6	0

**Figure 6:**
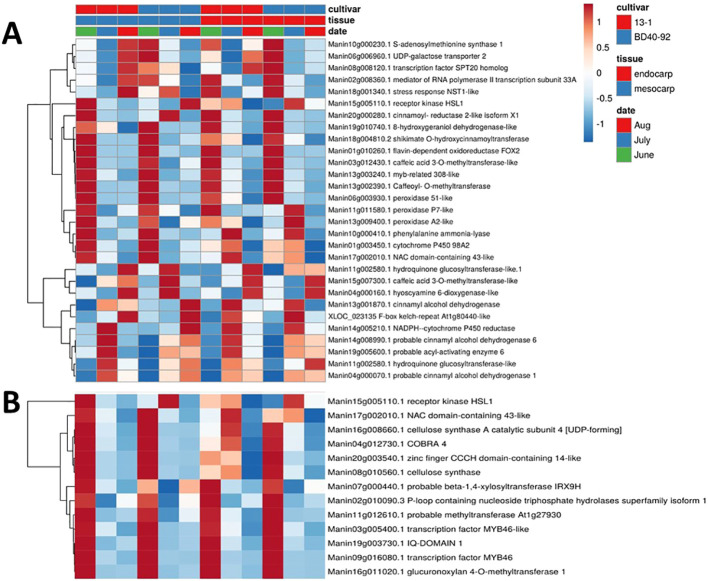
Ordered heat map of **(A)** 29 genes related to phenylpropanoid metabolic process/biosynthetic process and **(B)** 13 genes involved in plant-type secondary cell wall biogenesis and related processes in the mesocarp and the endocarp of the fibrous accession 13/1 and BD40/92. Expression levels were normalized by FPKM.

## 4 Discussion

In this study, we describe the morphological and gene expression patterns associated with mango fruit fiber development. While two types of fibers were identified - thick peripheral fibers beneath the fruit peal and fibers emerging from the seed (**Figure 1**) - our analysis focused specifically on the later type, which originate from the endocarp, and extend into the mesocarp. All mango fruits contain fibers, with “low-fiber” varieties characterized by shorter, thinner fibers and “high-fiber” accessions by longer, thicker fibers (**Figure 1**) (Ish-Shalom et al., 2023). Consumers generally prefer “low-fiber” mangoes; however, the biological basis of this trait has not previously been explored in mango.

The emergence of mesocarp fibers as a continuum from the fibrous “braids” of the endocarp (**Figure 2**, **Figures 3C, D**) suggests a shared developmental process. Both fiber types develop early in fruit development and harden and lignify in parallel during the seed hardening stage. However, in contrast to the endocarp, which is mostly composed of fiber and lignified structures, the mesocarp tissue contains mainly parenchyma cells, and only a small fraction of fibers (attempts to separate intact fibers for gene expression analysis were unsuccessful). Despite this, the PCA analysis of the gene expression patterns (**Figure 4A**) suggests that similar developmental processes occur in both mesocarp and endocarp tissues.

Gene expression analysis during fruit maturation and seed hardening indicates that lignin biosynthesis and secondary cell wall deposition are major processes involved in fibers hardening (**Figures 5B, C**). As fibers are present in both fibrous and non-fibrous fruits, genes associated with these processes were enriched in mesocarp and endocarp tissues of all accessions (**Figure 5**). Nevertheless, in the mesocarp of fibrous lines, an additional set of genes involved in lignin biosynthesis and secondary cell wall deposition was enriched, most of which were also expressed in the endocarp of the fibrous accessions (**Figure 6**). The expression of genes related to lignin biosynthesis and secondary cell wall deposition appears to be developmentally regulated, contributing to endocarp hardening and lignin precipitation within existing mesocarp fibers—structures that histologically extend from the endocarp—early in the season. This activity likely facilitates fiber formation across all accessions. Histochemical staining supports a more intensive and widespread lignin deposition within the cell walls of the more fibrous cultivars (**Figure 2**). This increased lignification likely reinforced the fiber matrix yielding the characteristically rigid fibers that reduce fruit quality. reduce fruit quality.

Our transcriptomic profiling revealed a highly coordinated molecular network that simultaneously governs secondary cell wall biogenesis, phenylpropanoid biosynthesis, and structural matrix remodeling across both fruit tissues during fiber development. The shared activation of core phenylpropanoid structural genes, including cinnamoyl-reductase 1-like (CCR1), cytochrome P450 84A1-like (CYP84A/F5H), and multiple peroxidases (PER) and β-glucosidases, underscores a robust metabolic commitment to lignin synthesis and polymerization within the endocarp-mesocarp continuum. This active lignification is tightly coupled with targeted secondary cell wall deposition, as evidenced by the prominent expression of WALLS ARE THIN 1 (WAT1)-like genes, which are associated with the regulation of secondary wall formation in plant fibers (Ranocha et al., 2010), alongside chitinase-like proteins that are known to contribute to cellulose biosynthesis within developing vascular tissues (Aktar Hossain et al., 2010). Furthermore, the detection of co-expressed germin-like proteins points to an active mechanism for generating the hydrogen peroxide substrate required to drive the peroxidase-catalyzed cross-linking of these emerging cell wall components (Carter and Thornburg, 1999). Crucially, the structural maturation of these fiber strands appears to rely on a dynamic developmental balance between rigid polymer deposition and localized wall matrix remodeling or loosening. This requirement is clearly reflected in the synchronized regulation of pectin-metabolizing enzymes (including pectin acetylesterases, pectinesterases, pectate lyases, and polygalacturonases) that facilitate targeted pectin degradation and wall loosening (Gallego-Giraldo et al., 2020), alongside primary and secondary cell wall-restructuring enzymes such as xyloglucan endotransglucosylase hydrolases (XETs) and the xylan biosynthesis-associated IRX15-LIKE gene (Jensen et al., 2011; Kushwah et al., 2020). When integrated with the observed expression profiles of foundational structural cell wall proteins, such as extensins, required for cell wall self-assembly (Lamport et al., 2011), expansins, functioning as wall-loosening agents (Sampedro and Cosgrove, 2005), and members of the hydroxyproline-rich glycoprotein (HRGP) superfamily of plant cell wall proteins (MacMillan et al., 2015). These findings demonstrate that mango fiber development is driven by a highly complex, coordinated developmental program that orchestrates structural synthesis, protein self-assembly, and matrix modification.

While it is a common knowledge that the mango mesocarp fibers are attached to the hard stone, our findings provide a concrete histological tracking and molecular transcriptomic dataset that explains how this structural continuum develops at the tissue and cellular levels. The mesocarp fibers originate early in development as extensions of the fibrous endocarp, forming a continuous structural network that undergoes co-lignification during seed coat hardening. The coordinated activation of lignin biosynthesis and secondary cell wall formation pathways in both tissues suggests a shared regulatory mechanism that governs fiber differentiation and rigidity. In fibrous accessions, the elevated expression of genes involved in phenylpropanoid metabolism and lignin polymerization, including PER, CCR1, and CYP84A-like genes, likely drives progressive lignin accumulation and secondary cell wall maturation, resulting in the highly rigid, lignified fiber networks that extend deeper into the mesocarp. Conversely, in non-fibrous cultivars, reduced activity of these pathways results in limited lignin accumulation, yielding softer mesocarp tissue.

Interestingly, while our transcriptomic analysis identified significant differential expression among a broad suite of downstream structural genes directly involved in phenylpropanoid metabolism, lignin biosynthesis, and secondary cell wall deposition, specific upstream key genes acting as definitive master regulators of this pathway—such as distinct MYB or NAC transcription factors—were not prominently detected. This absence suggests that the co-lignification of the endocarp-mesocarp continuum may be governed by a highly decentralized, subtle, or temporally transient regulatory network, or perhaps controlled at the post-transcriptional level. Consequently, further targeted molecular and functional studies remain essential to fully decipher the precise upstream biological control mechanisms directing fiber development in mango.

In many drupaceous fruits, hardening of the endocarp occurs as the fruit matures via secondary cell wall formation and lignification, a process conserved in drupes across different taxa (Dardick and Callahan, 2014; Khan et al., 2022). Although many plants have fibers as part of their fruit composition, structural fibers in fruits are relatively rare. Coconuts have fibers in their mesocarp with a structure resembling mango fibers, but little is known about their biological origin (Beveridge et al., 2022). It has been suggested that in coconut, the fibers serve to protect the seed from mechanical disturbance. The function of the mango fibers, and their biological role remains unclear. They may serve to protects the seed from damage by herbivores. Interestingly, in the drupe fruits of the Rosaceae family (**Supplementary Figure 4**), some appear to have fibers connected to the endocarp. This is also been supported by the Clingstone and Freestone phenotypes peach and plums: Clingstone fruits, in which the flesh adheres to the stone, and Freestone fruits, in which the endocarp is not attached to the mesocarp (Wang et al., 2024). We therefore propose that mesocarp fibrousness reflects the developmental and molecular coupling between endocarp hardening and mesocarp fiber lignification, a process potentially conserved across drupaceous fruit species. Understanding this continuum offers new insight into the evolution of fruit structure in mango and provides a mechanistic basis for future efforts to breed varieties with reduced fibrousness.

Currently, no stoneless or freestone mango accessions are known. It is plausible that a mango without an endocarp or partial endocarp (i.e., a “stoneless” mango) would also lack structural fibers in the mesocarp. While no mango variety entirely lacks an endocarp, certain varieties like ‘Nom Doc Mai’, and ‘Noa’ have a very thin and flexible endocarp and are very low in fibers. In the Rosaceae family, stoneless plums (retaining only traces of endocarp) have been documented (Burbank, 1914; Callahan et al., 2015), and stoneless cultivars of Chinese Jujube were also developed and characterized (Mao et al., 2024). Similarly, in palm oil, a single mutation in the *SHELL* gene, homologous to *AGAMOUS*-like genes, can eliminate the endocarp (Singh et al., 2013). Although the knowledge of endocarp development in mango is limited, findings from other drupe fruits suggest that it may be possible to develop stoneless mangoes using biotechnological approaches. Alternatively, the rigidity of the endocarp could serve as an easily monitored trait for classical breeding to generate varieties with very low fiber content.

## Data Availability

The sequencing data from this study have been deposited in The National Center for Biotechnology Information BioProject database (https://www.ncbi.nlm.nih.gov/bioproject/) under the accession number PRJNA1336059.
