# Supplementary material for: Mango mesocarp fibers are a continuum of fibrous endocarp structures that are co-lignified during seed coat hardening
Source: Front Plant Sci. 2026 Jul 23;17:1874130. doi: 10.3389/fpls.2026.1874130 (PMC13441945; doi:10.3389/fpls.2026.1874130)
Supplement: Supplementary file 1 [file DataSheet1.docx]

Supplementary Material

# 1 Supplementary Figures and Tables

## 1.1 Supplementary Figures

**Supplementary Figure S1:** Schematic representation of fruit's region used for endocarp and mesocarp tissue collection for histological and molecular analyses. Fruits of different accessions at different developmental stages were cut open into halves. The developing embryo (Emb) were and the fruit peel (Peel) were removed. For histological analysis, samples containing both the mesocarp and the endocarp from the base of the ventral side of the fruit were collected as indicated by the Dasshed black lines. For the molecular analysis, the mesocarp and the endocarp of the regions were collected separately.

**Supplementary Figure S2.** Differentially expressed genes (DEGs) of the mesocarp & endocarp tissues during seed hardening for four selected accessions.

**Supplementary Figure S3.** Kobas Go analysis of 492 differentially expressed genes. Corrected P ˂0.05. Four categories of cell wall and lignin associated genes are marked in red boxes.

**Supplementary Figure S4.** Fibers penetrating from the endocarp into the mesocarp in stone fruits (*Prunus* species): (a) Fibers emerging from the stone of a plum into the mesocarp tissue (white arrow heads). (b) Fibers emerging from specific sites in the stones of peach fruit toward the fleshy mesocarp tissue. Fibers were visualized following washing and removal of mesocarp tissue under running water, as described in Materials and Methods.

**Supplementary Table S1**

Summary of transcriptome sequencing of endocarp and mesocarp samples during development and seed hardening of four mango accessions and percentage of mapping to the 'Tommy Atkins' genome draft (Bally et al., 2021). The sequencing data from this study have been deposited in The National Center for Biotechnology Information BioProject database under the accession number PRJNA1336059.

**Supplementary Table S2:**

DEGs with common expression patterns in all accessions (fibrous and non-fibrous) and in both mesocarp and endocarp tissues during fruit development. (A). Expression profiles of 492 DEGs showing shared patterns across accessions and tissues. (B) Subset of 51 DEGs associated with secondary cell wall metabolism, phenylpropanoid biosynthesis, and related processes. Cluster analysis of the two sets of genes is provided in Figure 7A,B.
